# Updating Relatives of Patients Lacking Capacity in General Medicine: A Quality Improvement Project in a District Hospital in the United Kingdom

**DOI:** 10.7759/cureus.72369

**Published:** 2024-10-25

**Authors:** Krishnakanth Puneeth Khasnavis, Jegadis Sreeneyasan, Divya Kanakalingam, Aishwarya Krishna Kumar

**Affiliations:** 1 Internal Medicine, Royal Albert Edward Infirmary, Greater Manchester, GBR; 2 Medicine, Royal Bolton Hospital, Bolton, GBR; 3 Medicine, Royal Liverpool University Hospital, Liverpool, GBR; 4 Medicine, Hinchingbrooke Hospital, Cambridgeshire, GBR

**Keywords:** communication in healthcare, deprivation of liberty safeguards, family update, general internal medicine, quality improvement, relative update

## Abstract

Background

Patient-centred care involves focussing on the individual’s needs. Where patients lack capacity, it is important to involve relatives to better understand their needs, encourage positive healthcare outcomes and provide good quality care. Cultivating good rapport between clinicians and families also improves patient safety and satisfaction.

Aim

This project aims to improve the process of updating relatives regularly within various medical departments in the hospital. The quality improvement (QI) also strongly advocates for a minimum of two updates within a week of admission, for patients who lack capacity and who are on Deprivation of Liberty Safeguards (DOLS). This framework can be extrapolated to different medical settings to ensure ongoing patient care is conveyed and discussed effectively with families at regular and frequent intervals.

Methodology

This was a retrospective study that involved a total of 121 patients who lacked capacity and who were admitted to the acute medical unit, cardio-respiratory wards and geriatric wards in a district hospital. The project was designed using Plan-Do-Study-Act (PDSA) cycles and conducted over four months. Data mainly focused on details of relative updates from the time of admission, which were extracted from electronic records. Two interventions were conducted, with data gathering done before and after each intervention to ensure completeness of each PDSA cycle and measure the efficacy of the intervention. The first and second audits involved 56 and 65 patients, respectively.

Results

Data were collected regarding the number of relatives being updated within the first 48 hours and first week of admission. Collected data also involved details of the staff involved in these updates and the content of the updates. Clinicians accounted for the larger proportion of the staff conducting relative updates for patients on DOLS. Five (36%) in the first audit and 8 (37%) in the second audit of acute medical wards saw doctors at various training levels and roles carrying out the relative updates. In Cardio-Pulmonary wards, these numbers were 50% (8) in Audit 1 and 44% (7) in Audit 2. The greatest clinician burden was observed in geriatric wards wherein 73% (19) in Audit 1 and 53% (15) in Audit 2 of relative update data showed clinicians performing these updates.

Coming to the frequency, collated data show an overall positive trend across all the wards where relatives were updated within one week. A positive trend was noted, especially in the Geriatric and Cardio-Pulmonary wards, with improvement in the first week of updates going up, from 35% to 46% and 29% to 48%, respectively.

Significant room for improvement in updating relatives within 48 hours of admission still exists. About 53% of the updates included information about the patient's condition. Details regarding follow-up were only noted in 41% of the updates.

Conclusions

This study highlights the need to ensure that the relatives of patients who lack capacity are updated more regularly. Colleagues are encouraged to use appropriate documentation methods such as relative progress notes to ensure ease of future updates. It is essential that relatives are updated not only on the patients' condition and diagnosis but also, whenever possible, on investigations, management, and follow-up. A minimum of two updates in the first week is highly advisable. Effective communication and regular updates improve discharge planning and patient outcomes.

## Introduction

Deprivation of Liberty Safeguards (DOLS) is an effective safeguarding tool that deals with patients who do not have the capacity to make independent and rational decisions regarding their health, treatment and well-being [1]. DOLS is an essential human rights mechanism and aims to serve only in the best interests of the patient involved. Although this means continuous monitoring and control, DOLS is only enforced when it is necessary and works towards the well-being of the individual [1].

As life expectancy continues to gradually rise due to better treatment options and medical ventures, the geriatric population are usually the most common occupants of medical wards across the United Kingdom. Infections and other medical conditions predispose them to a state where DOLS is warranted for their safety and well-being. Updates regarding these patients to their families are vital, as the patient is unable to communicate hospital events and because treatment options must be framed in accordance and coalition with the family. 

Across the United Kingdom, there are mounting A and E waiting lists with overwhelming wait times for beds in acute wards. This translates to a very busy ward with very little time to perform tasks such as updating family. This is then overlooked in most instances and patients’ relatives are unfortunately left not updated for several days to weeks, leading to confusion and worry. The primary aim of this quality improvement plan (QIP) was, therefore, to ensure that relative updates were done regularly in patients with DOLS.

## Materials and methods

This study was a retrospective analysis of six medical wards in a District General Hospital (DGH) in Wigan, United Kingdom, from the duration of August 2023 to October 2023. Local approval was sought and obtained from the Audit Team before the commencement of the QIP.

Inclusion criteria involved all patients regardless of age, who were lacking capacity and who had been admitted to the medical wards. Data were gathered from the uniform local electronic system (Health Information System, or HIS) that is used throughout the hospital. Two time frames were selected - within 48 hours and within the first week - to understand the disparity between both.

First intervention

The area of improvement identified was that the relatives of patients who were on DOLS were not being updated regularly. This could be rectified for better patient outcomes and to encourage patient-centric care along with support from families.

**Figure 1:**
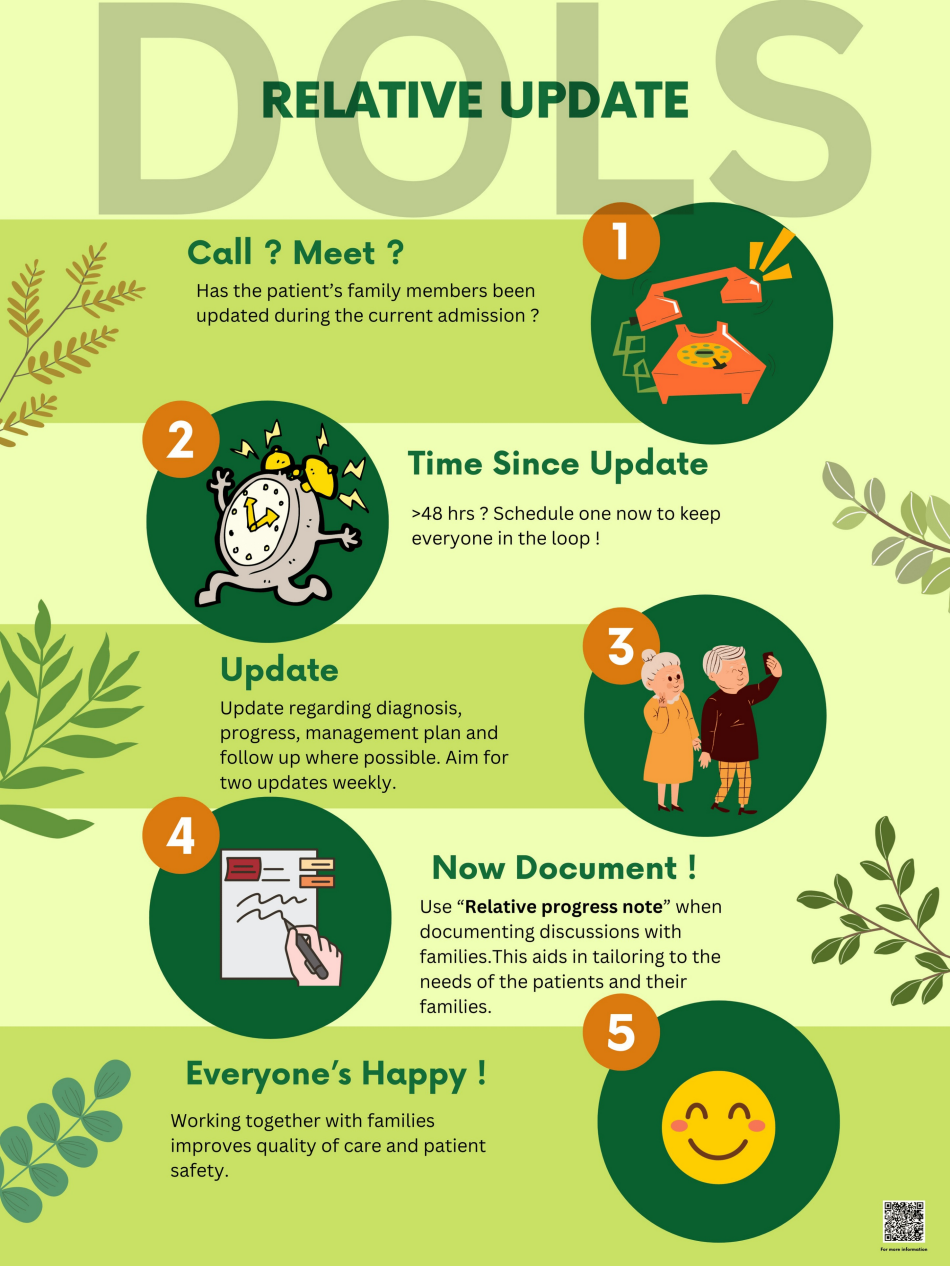
Poster: Guide to effective communication with relatives of patient on DOLS. Image credit: Krishnakanth Puneeth Khasnavis and Aishwarya Krishna Kumar. DOLS, Deprivation of Liberty Safeguards

The initial intervention consisted of creating a poster as shown in Figure 1. The posters were kept in easily accessible and noticeable places across all six medical wards. Additionally, these posters were also displayed in frequently visited areas of the hospital such as the hospital mess and canteen. The information regarding the poster was also conveyed to the ward managers of the respective wards. The poster was first vetted by a small focus group comprising junior doctors to gather feedback and suggestions for improvement. This intervention was aimed to help spread awareness and advice for a uniform DOLS framework to be adopted.

First audit

After a brief period following the first intervention, data were obtained from all the medical wards. These data were reviewed to select patients who fell into the inclusion criteria. Information was then collected by observing if there had been any updates to the family in the first 48 hours and the first week of admission. The contents of the update were also scrutinised to see if they fulfilled or included the following - Condition, Diagnosis, Management and Follow-up.

Second intervention

The second intervention comprised in-person teachings at the Acute Medicine Department and other departmental meetings. This enabled for a targeted introduction of the current QIP aims and objectives to the medical staff. Although the posters had been noted by many, the teaching further helped to reinforce the proposal for a minimum of two updates in the first week of admission for DOLS patients. 

Second audit

Following the second intervention, another data collection and review was conducted using the same wards to look for further improvement. The data were then thoroughly analysed and compared to the results from the first cycle.

## Results

The first audit involved gathering data from 56 patients, while the second audit had 65 patients who lacked capacity and who were on DOLS. Due to the study's purpose and the nature of patient admissions, the wards were divided into three categories - Acute Medical, Geriatric and Cardio-Pulmonary - each consisting of two wards. The median age of male and female patients in the acute medical wards was 82.6 and 84, respectively, for Audit 1, and 83.6 and 84 for Audit 2. The median age of male and female patients in the Geriatric and Cardio-Pulmonary wards for Audit 1 was 83.6 and 83, and 77 and 80, respectively. For Audit 2, the median ages were 80 and 80, and 83 and 85.5, respectively.

**Figure 2:**
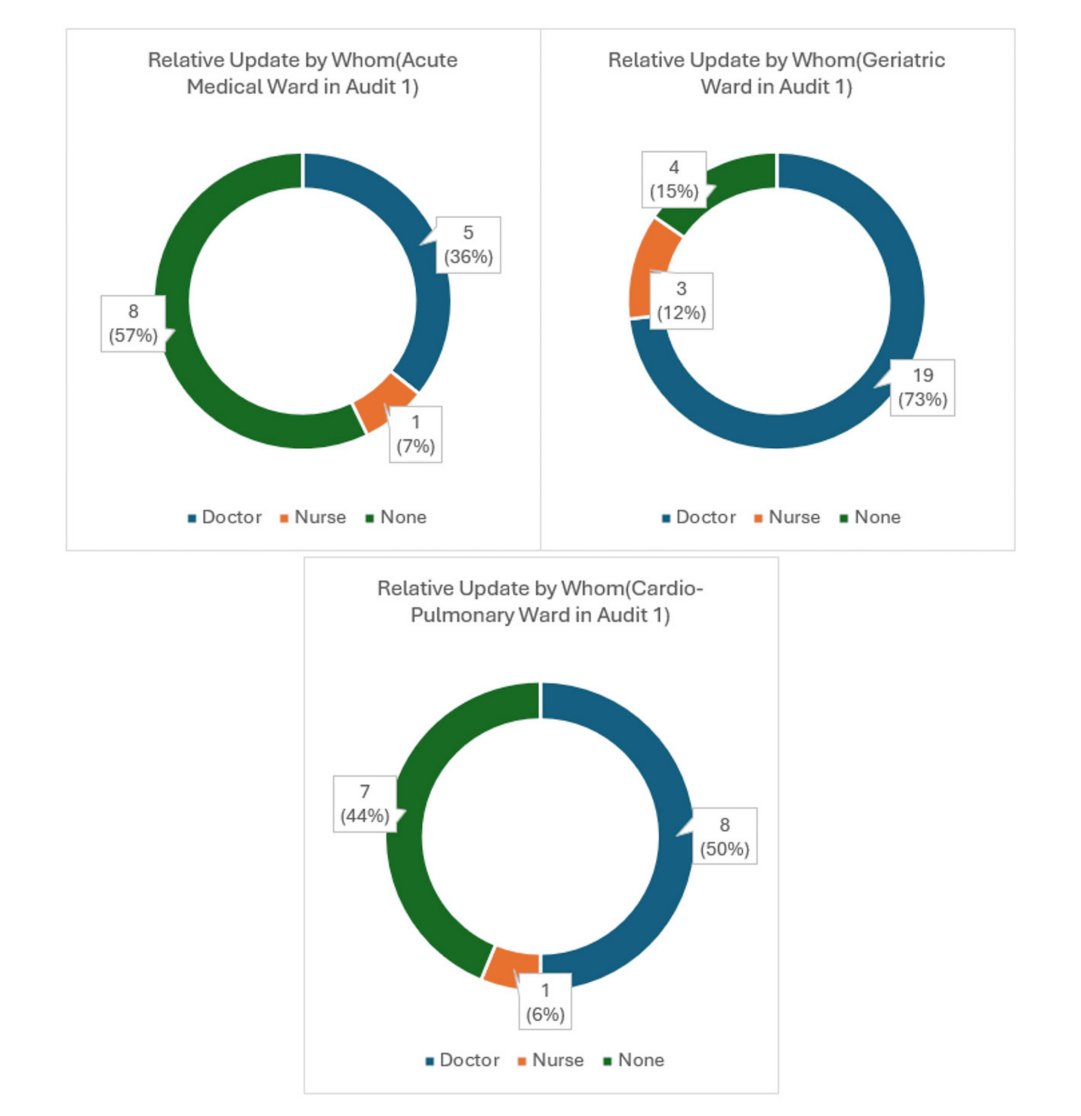
Relative update in Audit 1.

**Figure 3:**
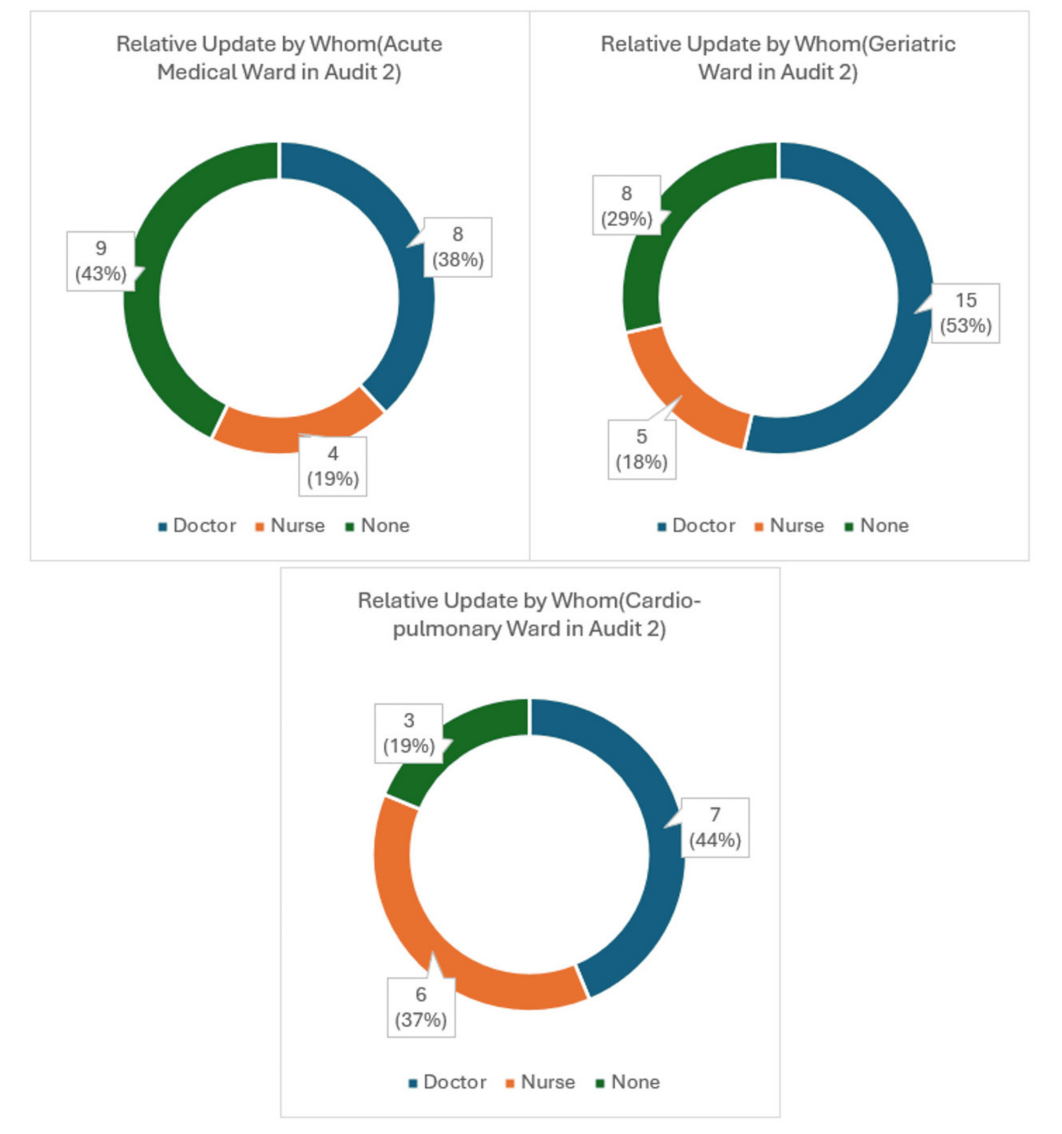
Relative update in Audit 2.

Notably, most updates were conducted by clinicians, as reflected in both audits (Figures 2-3). In Audit 1 of the Acute Medical wards, 36% (5 patient relatives) were updated by clinicians, 7% (1 relative) were updated by nursing staff, and the remaining 57% (8 patient relatives) were not updated (Figure 2). This trend had improved slightly in Audit 2 wherein 38%, or 8, patient relative updates had been conducted by clinicians, 19%, or 4, relative updates done by Nursing staff, leaving 43%, or 9, relatives not being updated (Figure 3).

In the Geriatric wards, the general trend of conducting relative updates along with the clinician burden seemed to be higher as compared to the Acute Medical wards. In Audit 1, 73%, or 19, relative updates had been done by doctors, 12%, or 3, by nursing staff and only 15%, or 4, relatives were not updated (Figure 2). This number remained high when compared to both the Acute Medical and Cardio-Pulmonary wards in Audit 2 where 53%, or 15, relative updates were undertaken by clinicians, 18%, or 5, by nursing staff and no update in 29%, or 8, patient relatives were noted (Figure 3).

Finally, in Audit 1, the Cardio-Pulmonary wards reflected data, with 50%, or 8, relative updates done by clinicians, 6%, or 1, by nursing staff and the remaining 44%, or 7, with no updates (Figure 2). This percentage ratio remained similar, with no significant change in Audit 2, where 44%, or 7, patient relative updates were performed by clinicians, 37%, or 6, by nursing staff and 19%, or 3, with no updates (Figure 3).

**Figure 4:**
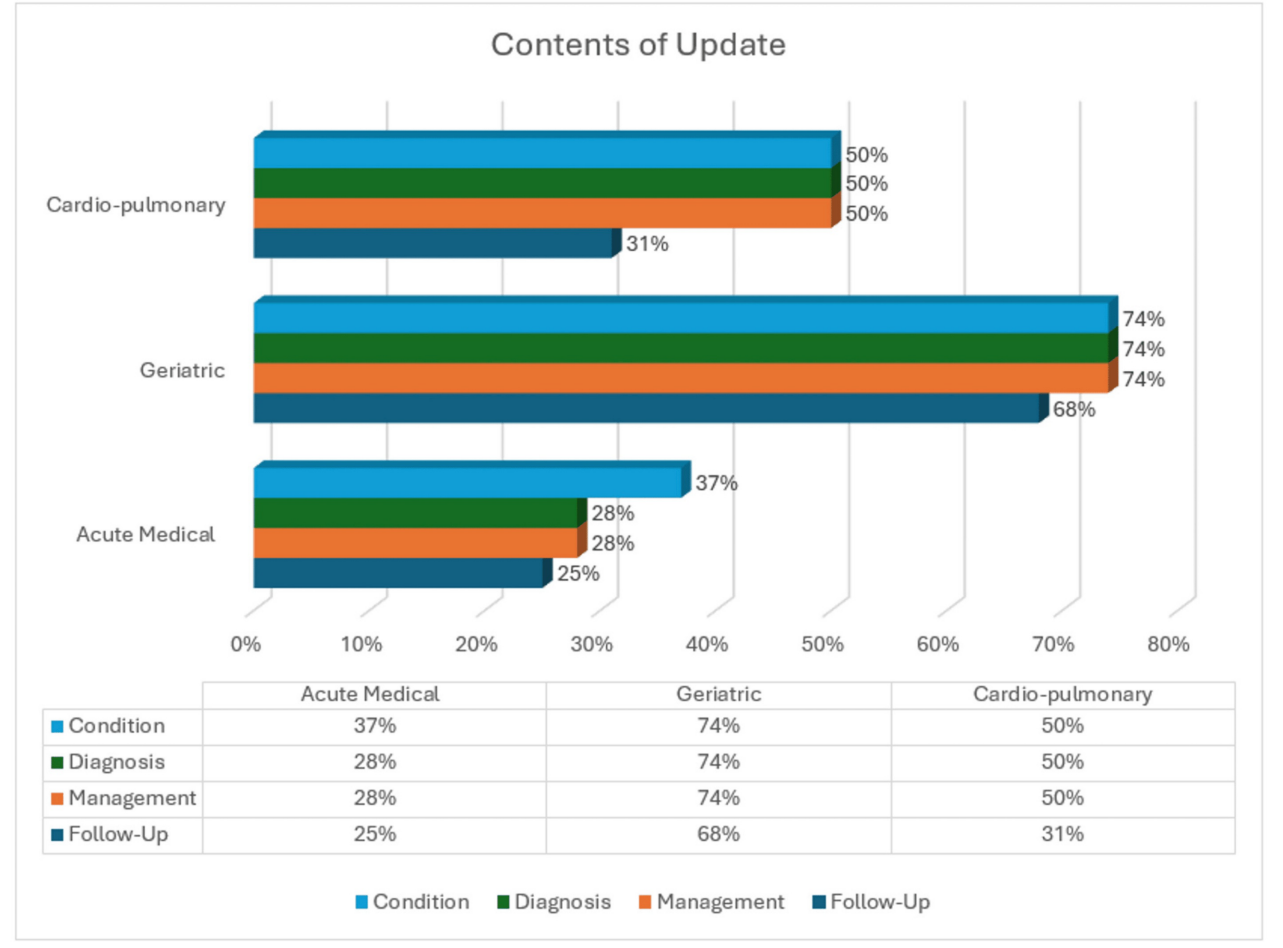
Content of updates: Condition, Diagnosis, Management and Follow-up.

Data regarding the content of the relative updates yielded higher percentages in terms of condition, approximating 53% across the six wards. The diagnosis and management were not conveyed to the relatives/family efficiently, with an average of 50% for both. The updates regarding follow-up were found to be either inconclusive or poorly communicated. It averaged 41% across the six wards and received the lowest score among the different components of the update (Figure 4). The data skewness may be attributed to rapid bed turnovers or patients being transferred to different wards before management and follow-up plans are fully established. This problem is further exacerbated if the condition of the DOLS patient is still under investigation.

When examining individual divisions, the Geriatric wards outperformed the others, achieving a uniform 74% in updates regarding condition, management, and diagnosis (Figure 4). Of the 54 updates, 40 had a proper transfer of the aforementioned information. Sixty-eight per cent (37) of the relative updates in Geriatric Wards included the follow-up plan for the patients (Figure 4). Cardio-Pulmonary wards had 50%, or 16 of 32, updates detailing condition, diagnosis and management. Thirty-one per cent, or 10 of 32, updates mentioned follow-up. The acute medical wards had 37%, or 13 out of 35, updates discussing the condition, and 28%, or 10 out of 35, updates mentioning diagnosis and management. Twenty-five per cent, or 9 of the 35, updates had follow-up included in them (Figure 4).

**Figure 5:**
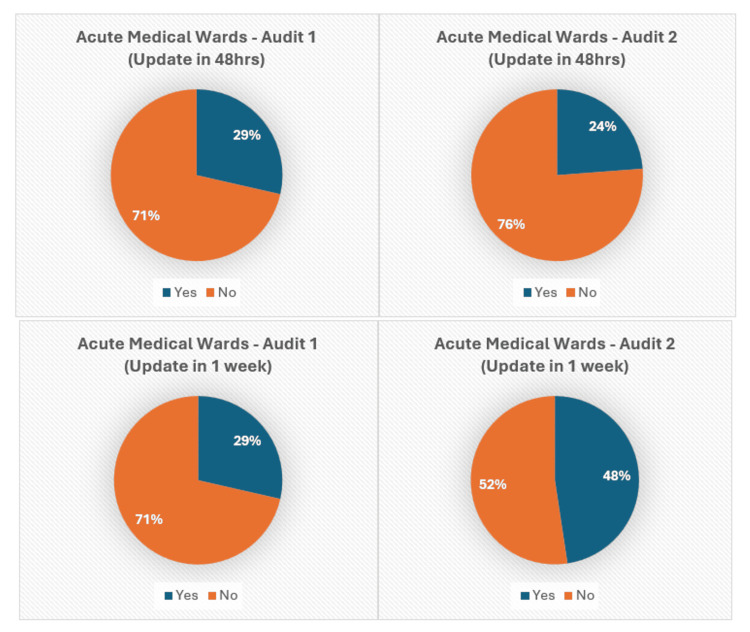
Relative updates in Acute Medical wards (48 hours, one week).

**Figure 6:**
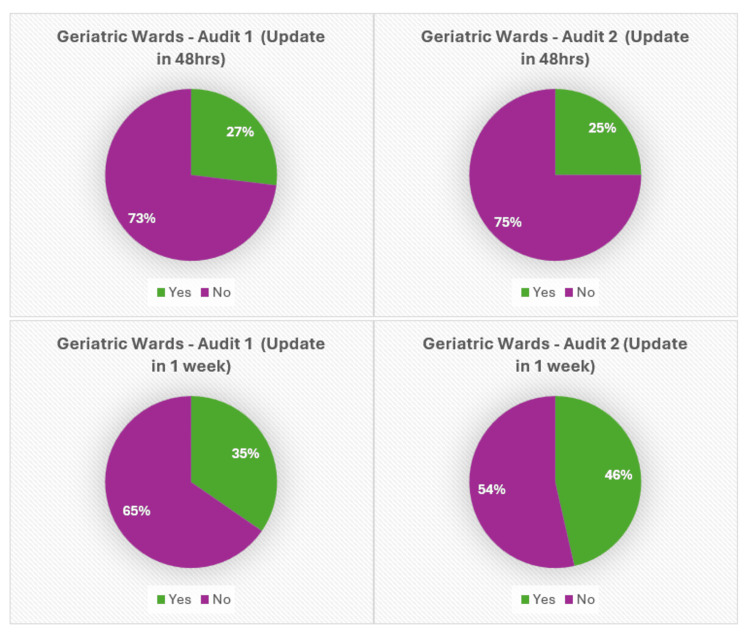
Relative updates in Geriatric wards (48 hours, one week).

**Figure 7:**
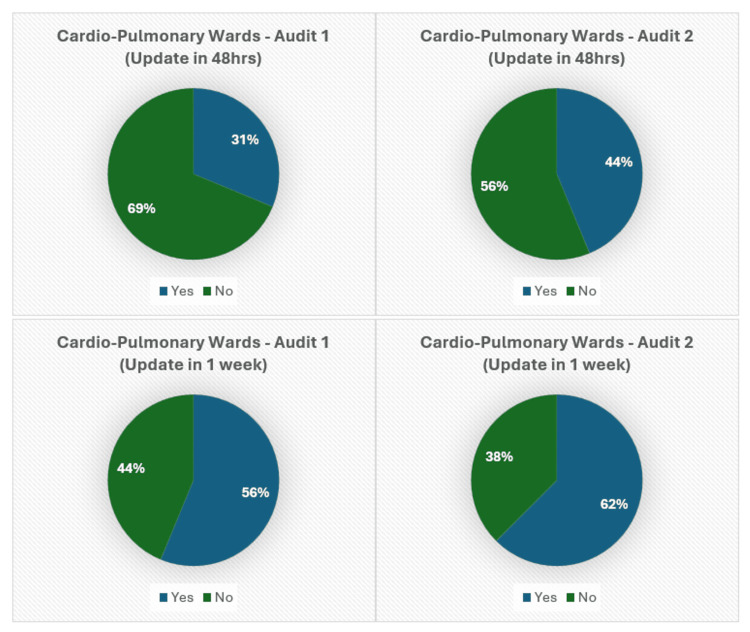
Relative update in Cardio-Pulmonary wards (48 hours, one week).

Figures 5-7 present the pie chart analysis of Audits 1 and 2, showing the number of patient relatives who were updated within 48 hours or the first week of admission.

First, it was noted that there was a sharp increase in relative updates performed within the first week as compared to within the first 48-hour time frame. After the intervention, there was a mixed response to the 48-hour target. However, there was a significantly positive trend with a steep increase in several relative updates performed within the first week of admission. In the Geriatric wards, relative updates increased to 46% from 35% (Audit 1: 9/26 noted, Audit 2: 13/28 noted), and in the Acute Medical wards, they increased from 29% to 48% (Audit 1: 4/14, Audit 2: 10/21) for the first-week target (Figures 5-6).

A mild improvement was noted for both the 48-hour and first-week targets in the Cardio-Pulmonary wards, with percentages increasing from 31% to 44% for the 48-hour updates (Audit 1: 5/16, Audit 2: 7/16) and from 56% to 62% for the first week (Audit 1: 9/16, Audit 2: 10/16), respectively (Figure 7).

A mild decline in the number of relative updates was observed for the 48-hour targets in both the Acute Medical and Geriatric wards, with the Geriatric wards decreasing from 29% to 24% (Audit 1: 7/26, Audit 2: 7/28) and the Acute Medical wards dropping from 27% to 25% (Audit 1: 4/14, Audit 2: 5/21) (Figures 5-6).

## Discussion

This QIP is aimed to enhance communication with relatives of patients under DOLS, ensuring that relatives receive at least two updates per week. The data revealed a positive trend in relative updates following the two interventions, reflecting their success.

Improving communication with relatives leads to better patient outcomes [2], higher family satisfaction and reduced distress during hospital admissions. Angood et al. explained how systems and care can improve significantly if patient and family inputs are considered and given weight to [3]. As patients and families are a limitless resource, they can help the overburdened healthcare system succeed in the long run.

Hospital admission can be intimidating and frightening for patients and families. Therefore, having support in these times is crucial, especially when having to make decisions that could impact the ongoing treatment and management. Patients on DOLS or who lack capacity require further assistance and support in this regard as they are unable to communicate their wishes and opinions. This vulnerable group of patients hence require a higher level of care, one that necessitates meticulous discussion with family members and relatives steering management towards what is in the best interests of the patient.

The role of family in patient care is more pronounced in critical patient scenarios and ICU admissions. Studies done in ICU settings have shown that families have expressed their interest in being part of the caring practices [4] and are motivated to provide support where needed, as this helps with their confidence in the patient care received [5]. While healthcare providers [6] and nursing staff [4] were receptive to family involvement in patient care, the creation of uniform policy guidelines has been recommended. The need for these guidelines arises partly from apprehension and negative family attitudes towards invasive procedures and resuscitation [6], as well as the necessity to preserve patients’ autonomy.

The project also emphasises how crucial the role of clinicians is in communication with families. In ICU and ward-based settings, patients and caregivers often lean towards collaborative group discussions rather than individual discussions with healthcare staff [7]. As patients under DOLS rely completely on families and clinicians to tailor and decide their treatment, the burden is far more difficult to shoulder for both members of the family and doctors. This further emphasises the dire need for the healthcare system and clinicians to integrate family into the treatment and management of patients, especially for those who lack capacity.

Family interests and opinions regarding treatment options are often lost due to many variable factors. Examples include changes in the parent medical team, lack of rapport and dynamic management plans without proper discussion with families. This highlights the need for clear documentation practices, such as using dedicated *relative progress notes* in the electronic health record (EHR) to streamline updates and ensure continuity in communication when patients are transferred between wards. Expanding the role of nursing staff in providing updates may also help alleviate the burden on clinicians in acute settings.

Several limitations to the current study have been identified. As this was a single-point study involving a single institution, data assimilated might be limited and may not represent challenges faced in other hospital settings. The high patient turnover and acute nature of the wards studied may have influenced the ability to provide timely updates, particularly within the 48-hour window. Additionally, the reliance on retrospective data from electronic health records may have introduced data inaccuracies or missed updates that were not properly documented.

Moving forward, several areas for further improvement can be identified. Involving other members of medical staff such as nurses and social workers in providing updates could distribute the responsibility more evenly. This can potentially reduce the burden on clinicians, especially in acute wards where time constraints are a common barrier. Additionally, expanding this QI initiative to other hospital wards or different hospitals would allow for a better understanding of the scale of this project. Lastly, future studies should aim to assess the long-term sustainability of these interventions by tracking relative update compliance over a longer period and evaluating their impact on family satisfaction and patient outcomes.

As families are stepping up to offer and provide informal care [8] for loved ones admitted to hospitals, the healthcare system must likewise adapt and augment this move by offering more transparency and support. Policies trialled locally and then implemented in other hospital and healthcare systems could help reinforce both the necessity of family to be integrated into patient care and to ensure relatives of patients on DOLS are being regularly updated.

## Conclusions

Communication is key, and some patients require tailored support with special requirements. It is, therefore, paramount to involve the relatives in patient management and decision-making from early on. Updating the family twice a week regarding the condition, diagnosis and progress is ideal. This can further be more productive if management and follow-up plans are also conveyed in the same setting.

Although it is a challenging situation, it can help promote transparency and aid relatives or family members to make informed decisions. Family engagement can help in discharge planning and improve the quality of the treatment provided to patients, especially for those who lack capacity. This QIP successfully demonstrated the importance of regular updates to relatives of patients under DOLS, leading to improvements in communication across multiple wards. Integrating this DOLS framework into the healthcare system will significantly reduce the stress felt by the relatives, ensure that the family members are well-informed and will improve all aspects of inpatient care.
